# Effect of a Shock Micro-Cycle on Biochemical Markers in University Soccer Players

**DOI:** 10.3390/ijerph18073581

**Published:** 2021-03-30

**Authors:** Diana García-Cardona, Patricia Landázuri, Oscar Sánchez-Muñoz

**Affiliations:** 1 Biomedical Sciences Program, Research Group on Cardiovascular and Metabolic Diseases (GECAVYME) and Research Group on Physical Activity and Health Physiology (GIFAS), University of Quindío, Armenia 63000, Colombia; 2 Medicine Program, Research Group on Cardiovascular and Metabolic Diseases (GECAVYME), University of Quindío, Armenia 63000, Colombia; plandazu@uniquindio.edu.co; 3 Sánchez-Muñoz OE, Physical Education and Sports Program, Research Group on Physical Activity and Health Physiology (GIFAS), University of Quindío, Armenia 63000, Colombia; oesanchez@uniquindio.edu.co

**Keywords:** biomarkers, soccer, oxidative stress, lipids

## Abstract

This study aimed to examine various biochemical biomarkers changes during a shock micro-cycle in soccer players from a university team. The study had 22 players (age: 22 ± 3 years; body mass: 68.6 ± 7.1 kg; height: 1.73 ± 0.07 m). The study measured total cholesterol (TC), triglycerides (TG), cholesterol linked to high-density lipoproteins (HDL), low-density lipoproteins (LDL), very low density lipoproteins (VLDL), arterial index (AI), creatine kinase (CK), glutamate-oxalacetate-transaminase (GOT), glutamate-pyruvate-transaminase (GPT), creatinine (Cr), catalase (CAT), superoxide dismutase (SOD), cytokines IL6 and TNFα, total antioxidant capacity (Cap antiox tot), hemolysis percentage and glomerular filtration rate (GFR); measurements were conducted during a shock micro-cycle. The lipid profile variables had no statistical significance when compared on day 1 with day 14. Except for TNFα, the other biomarkers compared with day one had progressive increments until day seven, with a subsequent reduction on day 14; however, none of the biomarkers returned to baseline values despite this decrease. The data shown herein suggest the need to research these biomarkers in distinct types of mesocycles, exercise, intensity, load, and duration to diminish fatigue and improve athlete performance.

## 1. Introduction

Soccer players are subject to an organizational structure of their training called mesocycle, within which the shock micro-cycle can be found, which is characterized by a large overall volume of work and a high level of incitement; its purpose is to stimulate the processes adaptation of the organism [1]. The high load of physical exercise and psychological stress can trigger an imbalance between the production of reactive oxygen species and other free radicals and the antioxidant defense mechanisms of the organism. This can cause molecular damage that can be measured through: biological markers, such as proteins; the activity of enzymes or their metabolites; lipid peroxidation; and nucleic acids, among others [2,3]. Several of these biomarkers have been studied concerning exercise, for example, biomarkers of: cardiac hypoxia [4], the immune system such as immunoglobulins [5], tissue damage or muscle activity [6], and some enzymes of the Redox system (catalase, peroxidase) [7].

Therefore, several biochemical markers could be used as indicators of physical stress, systemic inflammation, muscle damage, and physiological adaptation to sports activity [8]. Among the biomarkers that can be found, creatinine is a final product of muscle metabolism [9] and its activity (CK) is related to the intensity of the load to which the muscle is subjected [10]. In addition, its serum concentration is higher in athletes than in the population of people who are not physically active. Glutamate-oxaloacetate transaminase (GOT) and glutamate-pyruvate transaminase (GPT) serve as markers of liver disease. However, they could also be valuable tools to assess people’s metabolic responses to exercise [11].

Regarding inflammation, muscle damage initiates a local inflammatory response that involves the production of cytokines [12] such as IL6 and TNFα. In soccer studies such as that of Souglis et al. [13], increases were detected in IL-6 and TNF-α between two and four times higher than the values at rest, with the maximum values being those obtained immediately after the game. Regarding the lipid profile, specifically high-density lipoproteins (HDL), it is considered a protective factor [14] due to its role in the reverse transport of cholesterol. It is also described how exercise increases this lipoprotein.

In soccer during matches, players perform a large number of high intensity non-linear activities based on running [15]. In addition, acceleration and deceleration during direction changes produce high levels of mechanical and metabolic stress, which increases the demands of metabolic power and total energy expenditure during training and matches [16]. Specifically, they are necessary between 48–72 h to restore metabolic alterations after a match [17,18], although 3–4 days of recovery between successive matches may be insufficient to restore homeostasis [19].

Commonly, most of the studies investigating biochemical markers in soccer have been done through follow-up in one game, three games, or under the development of tests. In Colombian university soccer, specifically in the university zonal competitions, they play between three and five games with a short recovery time (about 24 h between matches). In this context, there is little information about it in the formal bibliography that studies and analyzes the behavior of said markers in Colombian college soccer. In this sense, the purpose of this study was to examine changes in various biochemical biomarkers during a shock micro-cycle in a university soccer team.

## 2. Materials and Methods

### 2.1. Subjects

A total of 22 (mean ± SD: age, 22 ± 3 years; body mass, 68.6 ± 7.1 kg; height, 1.73 ± 0.07 m) male students of the Universidad del Quindío soccer team took part voluntarily in this trial. These students train about 10 to 14 h per week for regional competitions. The study excluded athletes with diseases proven through their clinical history and excluded injured athletes and those with less than one year of training in the sports discipline.

### 2.2. Ethics

This research was carried out according to the Helsinki Declaration and Resolution 8430 by the Colombian Ministry of Health and Social Protection, and it was approved by the Bioethics Committee of the Physical Education Program at Universidad del Quindío.

### 2.3. Variables

The studied variables were divided into three groups: biochemical, anthropometric, and physical condition.

Biochemical: total cholesterol (TC), triglycerides (TG), cholesterol linked to high-density lipoproteins (HDL), low-density lipoproteins (LDL), very low density lipoproteins (VLDL), arterial index (AI), creatine kinase (CK), glutamate-oxalacetate-transaminase (GOT), glutamate-pyruvate-transaminase (GPT), creatinine (Cr), catalase (CAT), superoxide dismutase (SOD), cytokines IL6 and TNFα, total antioxidant capacity (Cap antiox tot), hemolysis percentage and glomerular filtration rate (GFR).

Anthropometric: age, mass, height, body mass index (BMI), adipose percentage, and muscle percentage.

Physical condition: maximal oxygen uptake (VO_2max_).

### 2.4. Procedure

The subjects participated in a 7-day shock micro-cycle.

Blood sampling: a blood sample was collected by venipuncture in a dried tube. Samples on days 1 and 14 were collected in the laboratory. Samples on days 4 and 7 were collected on the soccer field. Then, they were held in a portable freezer and transported to the laboratory. The serum was obtained through a centrifuge at 1000 g for 15 min, at 4 °C, separated in 1.5 mL micro-tubes, and stored until use (within two days).

Four blood samples were taken in the following manner:

Day 1: The blood sample was collected after 48 h without exercise and 12 h of fasting.

Day 4: The blood sample was collected once the training ended. Test subjects were under no fast conditions.

Day 7: The blood sample was collected once the training ended. Test subjects were under no fast conditions.

Day 14: Seven days after finishing the micro-cycle. The blood sample was collected after 48 h without exercise and 12 h of fasting. The measures of the 14th day aimed to determine if one week after of microcycle, biomarkers get the first day’s values. 

#### 2.4.1. Biochemical Measurements

The TC and TG were quantified via colorimetric enzymatic methods (Human^®^, Wiesbaden, Germany), HDL was assessed through initial selective separation with phosphotungstic acid/magnesium chloride (Human^®^), CK, GOT, GPT, and Cr were quantified via colorimetric enzymatic methods (Wiener Lab^®^, Rosario, Argentina), CAT and SOD were quantified via colorimetric enzymatic methods (Invitrogen^®^, Frederick, USA), cytokines IL6 and TNFα were determined through sandwich ELISA by reading the spectrophotometer results (Genesis 5) at 450 nm for both proteins following manufacturer’s instructions (BioLegend^®^, San Diego, USA), Cap antiox tot was quantified through the TBARS (Thiobarbituric Acid Reactive Substance) method, and hemolysis percentage was quantified via spectrophotometry.

The LDL, VLDL, and AI were calculated through formulas described for each.

The GFR was measured through the Cockcroft-Gault formula adjusted for body surface.

#### 2.4.2. Anthropometric Evaluation

Body mass was determined with the least clothing possible using a calibrated electronic scale (Tanita Bc-585f), and height was measured with a stadiometer (Seca Ref 216). To obtain the fat percentage, subcutaneous body fat was evaluated by using a skinfold caliper (Harpende) in six sites [20] (triceps, pectoral, subscapular, abdominal, suprailiac, quadriceps).

#### 2.4.3. Maximal Oxygen Uptake (VO_2max_)

The VO_2max_ was determined through the Course Navette (CN) test. The CN test was conducted based on the methodology described elsewhere [21]. Briefly, the participants were asked to run back and forth for 20 m, and in a straight line, and the running distance was defined by two cones at each end. The CN test ended when the participant stopped due to fatigue or when the participant did not reach the line defined before the beep signal. To determine VO_2max_, the equation proposed by Paradisis et al., [22] was used (Equation (1)).

(1) $$\mathrm{VO}2_{\max}(\mathrm{mL}/\mathrm{kg}/\min)=0.2761x+27.504$$

#### 2.4.4. Shock Micro-Cycle

The study subjects participated within their preparation period in a 7-day shock micro-cycle. Players did low-intensity training 72 h previous to this study. The micro-cycle was characterized by the increase in volume and intensity of the load. In such a way, micro-cycle development was carried out under the competition (regionals) simulation where the athletes had to perform for five games, the highest possible performance capacity. The characteristics of the micro-cycle are shown below.

Developer mesocycleShock Micro-cycle

Day 1: Smooth jog, joint movement, muscle activation, active stretching.
Day 2: Tactical training.
Day 3: Match 1.
Day 4: Match 2.
Day 5: Match 3.
Day 6: Match 4.
Day 7: Match 5.

For the soccer game, the coach formed two teams, which remains during microcycle with a 1-4-4-2 formation. A total of five games were played during the experimental period (one each day), all at the same time (08:30 h) on a natural grass surface. Each of the games lasted 90 min distributed in two halves of 45 min, and before each game they warmed up for 20 min.

The players were shown Borg’s perceived exertion scale upon ending each training session. All the subjects were familiar with this scale, as part of the regular monitoring of the training.

### 2.5. Statistical Analyses

Initially, a descriptive analysis was conducted in the laboratory of the results obtained from the biomarkers during the four days evaluated. Likewise, normality assumptions were verified. Then, a paired means test on the lipidic profiles was conducted to determine any difference in the measured variables on days 1 and 14. Additionally, a repeated measures ANOVA was performed, followed by the post-hoc test by Bonferroni. The sphericity assumption was verified through the Mauchly test. A *p*-value < 0.05 was considered as a statistical significance.

Analysis of the data obtained was performed with the SPSS software 22.0 (IBM).

## 3. Results

Table 1 shows the anthropometric and physical condition variables of the study subjects. It displays, concerning adipose and muscle percentage, that the athletes were within the adequate standards for age and sport; besides, the BMI is normal. Finally, the VO_2max_ was at an excellent level (46.50–52.40 mL/kg/min).

Table 2 shows the lipid profile results, and shows that the variables were within the values considered to be normal (TC < 200 mg/dL, TG < 150 mg/dL, HDLc > 40 mg/dL, LDL < 100 mg/dL, VLDL < 30 mg/dL, AI < 3.5 mg/dL) according to the ATPIII (Adult Treatment Panel III) [23]. Upon comparing these variables between days 1 and 14, no significant differences were evident despite their increase.

Table 3 presents the transaminase levels and hemolysis percentage. These variables had no statistically significant differences, although three variables (GPT, GOT, and hemolysis) increased progressively from days one to seven and decreased on day 14.

**Figure 1:**
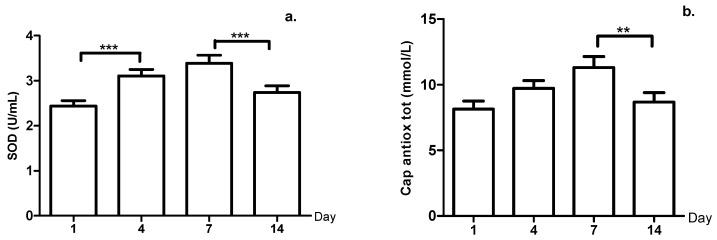
(**a**) Superoxide dismutase (SOD) levels. (**b**) Total antioxidant capacity (Cap antiox tot) during the micro-cycle, **: *p*-value < 0.01, ***: *p*-value < 0.001.

Figure 1 shows the levels of the oxidative stress biomarkers, showing that SOD and Cap antiox tot behaved similar to the prior variables, that is, with progressive increase from days one to seven. However, the SOD had significant differences from day one to day four (*p*-value = 0.001) (26.53% increase) and from day seven to day 14 (*p*-value = 0.001) (19.77% decrease), while in the Cap antiox tot, the decrease was only significant (23.36%) from days seven to 14 (*p*-value = 0.01).

**Figure 2:**
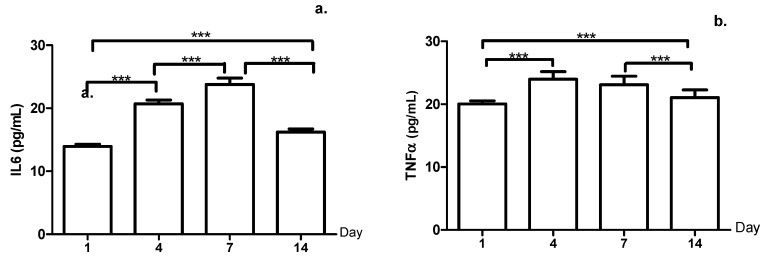
(**a**) Levels of Interleukin-6 (IL6); (**b**) levels of Tumor Necrosis Factor-alfa (TNFα) during the micro-cycle, ***: *p*-value < 0.001.

Figure 2 displays the IL6 and TNFα, with respect to IL6, with statistically significant differences in all days evaluated, while the TNFα had no differences between decreased concentrations from days four to seven (*p*-value = 0.068).

**Figure 3:**
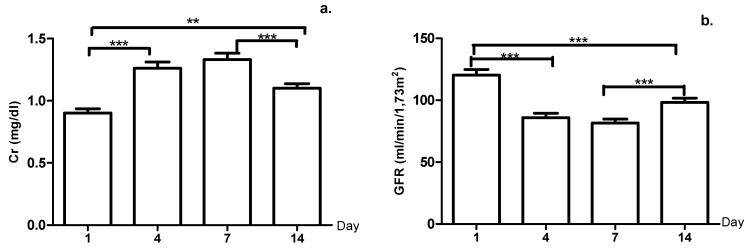
(**a**) Creatinine (Cr) levels; (**b**) Glomerular filtration rate (GFR) during the micro-cycle, **: *p*-value < 0.01, ***: *p*-value < 0.001.

For creatinine (Figure 3a), significant differences were evident, except for the increase from days four to seven (*p*-value = 0.801). Moreover, the GFR (Figure 3b) showed a decrease, from days one to seven, finding days four and seven with values (85.91 and 81.68 mL/min/1.73 m^2^, respectively) below the value considered normal (≥90 mL/min/1.73 m^2^).

**Figure 4:**
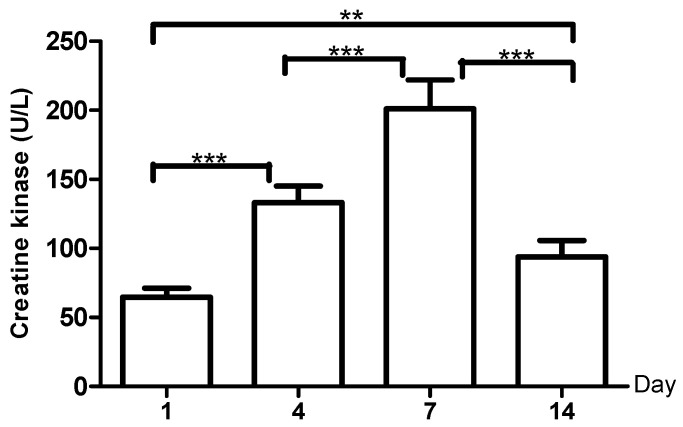
Creatine kinase levels during the micro-cycle, **: *p*-value < 0.01, ***: *p*-value < 0.001.

The CK (Figure 4) showed increased concentration (105.9%) from day 1 to day 4 (*p*-value = 0.001); from day 4 to day 7 (*p*-value = 0.001) (51.3%); and from day 7 to day 14 (*p*-value = 0.0002), a 53.37% decrease, all with statistical significance.

## 4. Discussion

This work studied some biochemical biomarkers during a shock micro-cycle of university soccer players.

Besides being subjected to academic stressors, the university soccer players were also subjected to other stressors due to their physical, technical, and tactical training to assume competition. High-level competitive sport requires the athletes to engage in high training loads, like a shock micro-cycle, which is characterized by a large overall volume of work and high level of incitement [1]. These high training loads and competitive stress produce changes in the body structure and metabolism to adapt to the physiological demands of training and competition [7].

The present study’s main finding was that the biochemical markers were affected by the micro-cycle, which was developed by simulating a college zonal competition. The effect was reflected in the increase in the athletes oxidative stress evaluated through SOD and Cap antiox tot. Likewise, the variation in the cytokines concentration, where the rise in IL6 (anti-inflammatory) appeared to influence the decrease in TNFα (pro-inflammatory), showed the anti-inflammatory effect of training. High muscle damage was also observed, evidenced by the increase in CK and Cr levels. Although there were variations in the level of transaminases during the micro-cycle, they were not statistically significant.

From the point of view of their body composition, soccer players constitute a differentiated population group due to their lower level of fat and higher muscle development. In this regard, this work found the BMI within the normal range [24] and percentage of body fat at 8.9%, that is, a good percentage for high-performance athletes. These results are similar to the elite soccer players reported in the study by Slimani et al., [25] and lower concerning the Greek soccer players (age 18–37 years) evaluated by Leão et al., [26]. The variable percentage of muscle mass was higher than that reported by García-Cardona et al. [27], who evaluated this same team in 2016 and found, on average, a percentage of muscle mass of 43.07%; in this respect, this average is consistent with results shown at fat percentage level, which was higher in 2016. The present study found similar values to that reported (46.18%) by Gil and Vedoy [28] in Spanish university soccer players. Concerning the VO_2max_ found in our study (51.66 ± 2.24), the result places them at an excellent level, which means they have an excellent physical condition for their sports performance.

The present study also measured lipid profile as a measurement of the athletes’ general health conditions; these variables were within the ranges considered normal for the lipid profile variables [23], as expected for individuals who practice a high-performance sport and take care of their health. These results are similar to those reported by Apostolidi et al. in Greek soccer players [29].

This work also assessed the hepatic function through transaminases; these enzymes indicate liver damage from disease or intake of medications or alcohol. The increase of these enzymes may be related to intense exercise [19]. Accelerated metabolic demands due to muscular exercise cannot be satisfied without a robust response by the liver [30]. In this work, transaminases increased on days four and seven of the shock micro-cycle; similar results were found by Nowakowska et al. [11]. Although these authors conducted an 11-month follow-up, and this work did so for only 14 days, the results show that on day 14 the transaminases had not returned to the baseline values of day one. In this regard, Pettersson et al. [31] state that intense muscular exercise can increase liver function at least seven days after exercise. In this same sense, Sjogren et al. [32] hold that individuals who engage in vigorous exercise (for example, weight lifters, marathon runners, and others] can have abnormal levels of transaminases due to a normal process of muscle tissue repair that causes inflammation and raises the levels of transaminases. The same authors indicate that transaminases can remain high for up to one week after stopping the exercise [32], as seen in the present research. Moreover, Chinedu et al., [33] also found increased GPT and other hepatic function markers, but not of GOT in soccer players subjected to training for 30 min, three times per week for three weeks. These findings suggest that vigorous exercise, like a shock microcycle affects transaminase levels.

It is known that exercise induces hemolysis [34] and although—at times—it is associated exclusively to impact sports, it is common in most sports modalities [35]. As potential sources of exercise-induced hemolysis, there are direct mechanical lesions caused by strong contact on the ground, repeated contractile muscle activity or vasoconstriction in internal organs, while pre-existing erythrocyte disorders or metabolic anomalies developed during exercise (for example, hyperthermia, dehydration, hypoxia, lactic acidosis, oxidative damage, increased catecholamine concentration) can contribute actively to triggering, accelerating, or amplifying this phenomenon [36]. It seems that the average life span of red blood cells in athletes is shorter, which is beneficial for performance [37]; different studies have identified significant hemolysis levels in long-distance runners and cyclists, among others. Our study also showed increased hemolysis on days four and seven of the micro-cycle without statistical significance; this non-significance likely is because, during the first phases of the exercise, hemolysis is stronger due to rapid destruction of the oldest population of erythrocytes, but these are renewed during the more advanced phases of training or competition, breaking down less, as shown by Yusof et al., in ultra-marathon runners [34].

Substantial evidence shows that exercise-induced hemolysis causes an increased inflammatory response and that it commonly occurs with increased IL6 [38], as with the results herein.

Although hemolysis could explain—in part—the increase of IL6, it is also known that exercise by itself can cause inflammatory states, such that IL6 and TNFα were also evaluated in this study. Cytokines are a diverse family of intercellular signaling molecules that regulate inflammation and immune response. According to Pussieldi et al. [39], intense acute exercise can induce muscle damage, producing cytokine release along with other local tissue factors related to the inflammatory phenomenon. In this study, IL6 levels increased continuously, while TNFα diminished as of day seven. In this respect, during exercise, muscle fibers release IL6 which also acts as a suppressor of TNFα production, like a pro-inflammatory cytokine [40]. The results agree with those reported by Stumpf et al., in elite soccer players [41], finding that after a stress test, pro-inflammatory cytokines IL-6, IL-8, TNF-a, and anti-inflammatory cytokine IL-10 were significantly high in all the soccer players.

Regarding the antioxidant capacity evaluated in this study during the shock micro-cycle, it was found that the CAT did not change significantly during the days considered. These results are similar to those reported by Jamurtas et al. [42] who assessed the effects of high-intensity interval training (HIIT) on the hematological profile and redox state in comparison with those following traditional continuous aerobic exercise. Although few indicators suggest that physical exercise provokes increased CAT activity in skeletal muscle, some studies describe a decrease or leveling of the catalase activity of the erythrocytes after exercising, [43].

Concerning the SOD enzyme, this is an important antioxidant defense that protects cells from ROS-induced oxidative stress [44]. The progressive increase of SOD found in this study was consistent with that reported by Silva et al. [45], who studied the biochemical impact of a sports season on professional soccer players and found that SOD increases, but then reaches its starting levels at the end of the season. Increased SOD can be explained by the increase in the superoxide radical during exercise. The superoxide radical is related to converting hemoglobin into meta-hemoglobin in erythrocytes during exercise and converted into H_2_O_2_ by the SOD enzyme [46]. Increased SOD in plasma could favor the accumulation of H_2_O_2_ and could indicate low efficiency of plasma elimination, which contributes to higher oxidative stress [47]. This is consistent with increased levels of lipid peroxidation (measured as TBARS) found until day seven in our study; during exercise, the process of supplying oxygen to active muscles can harm the polyunsaturated fatty acids in the membrane’s structures. In other words, it causes lipid peroxidation [48], and increased TBARS suggests that ROS production exceeded antioxidant activity [49] despite the possible adaptation. In this sense, Zoppi et al. [50] demonstrated that supplementing with vitamins C and E reduces TBARS levels; this supplementation with antioxidants could be preventing the adverse effect on the oxide-reduction balance generated by training in professional soccer players.

As part of the biochemical markers of oxidative stress in athletes, creatinine and the creatine phosphate enzyme were also measured. Creatinine is a product of creatine metabolism. Prior studies have indicated that athletes and active individuals with higher muscle mass, also have more elevated serum creatinine levels. The Cr concentration in this study increased from days one to seven and diminished on day 14 with significant differences on all the days evaluated. These results agree with those reported by Nowakowska et al. [11] and Ekun et al. [51], which show variations in Cr level over time. Likewise, studies, such as that by Jacobs et al. [52], mention that after moderate- or high-intensity exercise, there is diminished urine volume and a marked reduction in renal plasma flow and filtering rate that lead to increased Cr. This study also noted a decrease in GFR time up to day seven, which would support this hypothesis.

Concomitant with plasma creatinine variations, the same changes were noted in creatine phosphate levels, which increased from days one to seven and diminished on day 14. Among the biochemical markers considered important as indicators of metabolic changes or adaptations made during physical training, CK and its serum activity has been widely studied and is considered a marker of muscle damage [53]. Increased CK from days one to seven may be attributed to mechanical damage of the muscle cell membranes as a result of a substantial force of muscle contraction and oxidative damage of the cell membranes [54]. However, our results showed that seven days after ending the shock micro-cycle, and although the sample from day 14 had been taken with 48 h without exercise, the CK did not return to baseline values. The difference was statistically significant with respect to studies, like that by Wilk et al. [55] in subjects with 6.1 years experience in strength training, which shows how after 24 h of recovery there is increased CK activity, indicating severe damage to the muscle cell membranes and a CK leak in the bloodstream during exercise and after ending it.

Finally, it was observed that seven days after finishing the micro-cycle, IL6, TNFα, Cr, GFR, and CK did not return to baseline values and this difference was statistically significant, which demonstrates that seven days of recovery after five successive matches like the ones developed during the micro-cycle can be insufficient to restore the homeostasis.

As a later work, it is proposed to use these results to determine the relationship between the evaluated markers and the performance according to the playing position.

## 5. Conclusions

In conclusion, this study demonstrates that the soccer team athletes face stressors during training that produce significant changes in biomarkers related to muscle damage, inflammation, and oxidative stress. Still, some values return to normality when training intensity is lowered, while others remain high. These last factors could be used as fatigue or extenuation markers that could indicate to the coach the need for a more prolonged rest or of complementary measures to avoid severe harm or lesions in athletes who do not recover quickly.

The data shown herein also suggest the need to research these biomarkers in different types of meso-cycles, exercise, intensity, load and duration to diminish fatigue and/or improve the performance of the athletes.

## Figures and Tables

**Table 1:** Physical characteristics of participants.

Variables	*n* = 22
BMI (kg/m^2^)	23.66 ± 2.14
Body fat (%)	8.90 ± 0.93
Muscle percentage	46.84 ± 3.8
VO_2max_ (mL/kg/min)	51.66 ± 2.24

Mean ± SD. BMI: body mass index.

**Table 2:** Lipid profile.

VARIABLES	D1	D14	*p*-Value
TC (mg/dL)	153.80 ± 28.20	163.52 ± 32.3	0.495
HDL (mg/dL)	47.38 ± 7.10	48.55 ± 7.90	0.622
LDL (mg/dL)	88.58 ± 20.47	93.01 ± 21.56	0.666
VLDL (mg/dL)	16.66 ± 5.09	19.86 ± 4.67	0.101
TG (mg/dL)	78.62 ± 21.30	94.42 ± 21.49	0.051
AI	2.71 ± 0.84	2.90 ± 0.83	0.503

Mean ± SD. *n* = 22. D: day. TC: total cholesterol. HDL: high-density lipoprotein. LDL: low-density lipoprotein. VLDL: very low-density lipoprotein. TG: triglycerides. AI: arterial index. Note: No differences were observed among groups. Comparisons were tested via Student’s t-test.

**Table 3:** Biomarkers without statistically significant changes.

VARIABLES	D1	D4	D7	D14	*p*-Value	
GPT (U/L)	16.26 ± 3.2	17.49 ±2.30	21.27 ± 3.60	17.74 ± 3.50	D1 vs. D4	0.563
					D4 vs. D7	0.076
					D7 vs. D14	0.400
					D1 vs. D14	0.435
GOT (U/L)	19.50 ± 2.78	21.06 ± 3.10	24.37 ± 4.26	22.55 ±3.15	D1 vs. D4	0.586
					D4 vs. D7	0.086
					D7 vs. D14	0.290
					D1 vs. D14	0.296
Hemolysis (%)	80.24 ± 3.90	81.74 ±3.60	81.95 ± 2.54	81.25 ± 3.10	D1 vs. D4	0.204
					D4 vs. D7	0.833
					D7 vs. D14	0.429
					D1 vs. D14	0.107
CAT (U/mL)	28.64 ± 3.50	29.39 ±0.30	29.31 ± 0.26	29.4 ± 0.15	D1 vs. D4	0.341
					D4 vs. D7	0.403
					D7 vs. D14	0.217
					D1 vs. D14	0.345

Mean ± SD. D: day. GPT: glutamate-pyruvate-transaminase. GOT: glutamate-oxalacetate-transaminase. CAT: catalase.

## Data Availability

The data presented in this study are available on request from the corresponding author.
